# Protective Effects of White Button Mushroom (*Agaricus bisporus*) against Hepatic Steatosis in Ovariectomized Mice as a Model of Postmenopausal Women

**DOI:** 10.1371/journal.pone.0026654

**Published:** 2011-10-25

**Authors:** Noriko Kanaya, Makoto Kubo, Zheng Liu, Peiguo Chu, Charles Wang, Yate-Ching Yuan, Shiuan Chen

**Affiliations:** 1 Division of Tumor Cell Biology, Beckman Research Institute of the City of Hope, Duarte, California, United States of America; 2 Division of Bioinformatics, Beckman Research institute of the City of Hope, Duarte, California, United States of America; 3 Department of Pathology, Research Institute of the City of Hope, Duarte, California, United States of America; 4 Department of Molecular and Cellular Biology; Beckman Research institute of the City of Hope, Duarte, California, United States of America; The Chinese University of Hong Kong, Hong Kong

## Abstract

Nonalcoholic fatty liver disease (NAFLD) includes various hepatic pathologies ranging from hepatic steatosis to non-alcoholic steatohepatitis (NASH), fibrosis and cirrhosis. Estrogen provides a protective effect on the development of NAFLD in women. Therefore, postmenopausal women have a higher risk of developing NAFLD. Hepatic steatosis is an early stage of fatty liver disease. Steatosis can develop to the aggressive stages (nonalcoholic steatohepatitis, fibrosis and cirrhosis). Currently, there is no specific drug to prevent/treat these liver diseases. In this study, we found that white button mushroom (WBM), *Agaricus Bisporus*, has protective effects against liver steatosis in ovariectomized (OVX) mice (a model of postmenopausal women). OVX mice were fed a high fat diet supplemented with WBM powder. We found that dietary WBM intake significantly lowered liver weight and hepatic injury markers in OVX mice. Pathological examination of liver tissue showed less fat accumulation in the livers of mice on WBM diet; moreover, these animals had improved glucose clearance ability. Microarray analysis revealed that genes related to the fatty acid biosynthesis pathway, particularly the genes for fatty acid synthetase (*Fas*) and fatty acid elongase 6 (*Elovl6*), were down-regulated in the liver of mushroom-fed mice. *In vitro* mechanistic studies using the HepG2 cell line showed that down-regulation of the expression of *FAS* and *ELOVL6* by WBM extract was through inhibition of Liver X receptor (LXR) signaling and its downstream transcriptional factor *SREBP1c*. These results suggest that WBM is protective against hepatic steatosis and NAFLD in OVX mice as a model for postmenopausal women.

## Introduction

Non-alcoholic fatty liver disease (NAFLD) is the most common liver disease in adults in developed countries [1]. It is characterized by excessive hepatic lipid accumulation and is not related to alcohol use. NAFLD includes various hepatic pathologies ranging from hepatic steatosis to non-alcoholic steatohepatitis (NASH), fibrosis and cirrhosis. NAFLD is associated with metabolic syndrome, which has been characterized by obesity, type 2 diabetes, arterial hypertension, and hypertriglyceridemia in mice [1], [2]. Also, cirrhosis is a risk factor for the development of portal hypertension, hepatocellular carcinoma and liver failure [1]. It has been suggested that estrogen provides a protective effect on the development of NAFLD in women [3]. Therefore, postmenopausal women have a higher risk of developing NAFLD due to a lower level of circulating estrogen [3]. Due to an increased average life span, present generations of women can expect to spend at least a third of their lives in the postmenopausal state [4]. Dietary and lifestyle guidelines to reduce overall body weight may help avoid NAFLD, however, there are no specific drugs to treat this liver disease [5].

Mushroom has been reported to have many bioactive compounds (mycochemicals) [6], [7]. It has been reported that components of various species of mushrooms have a positive impact on human health through effects on the immune system, lipid levels in blood and liver, and tumor growth [6], [8], [9], [10], [11]. It is reported that several species of mushroom, including white button mushroom (WBM) (*Agaricus bisporus),* oyster mushroom *(Pleurotus Ostreatus)* and Shiitake (Lentinus edodes), reduce the cholesterol level in serum and/or liver [8], [12], [13]. Furthermore, recently mukitake mushroom (*Panellus serotinus*) was reported to alleviate nonalcoholic fatty liver disease in *db/db* mice [9]. However, the exact mechanisms to prevent NAFLD and its active components have not been identified yet. WBM constitutes 90% of the total mushrooms consumed in the US. Nevertheless, information is limited on health benefits of WBM [6]. Previous data in our laboratory showed that WBM had a significant inhibitory effect on the growth of the estrogen receptor (ER)-positive, aromatase-overexpressing breast cancer cell line, MCF-7aro. This effect was through the inhibition of aromatase, the enzyme that catalyzes the formation of estrogen from androgen. In addition, WBM intake inhibited MCF-7aro tumor growth in nude mice. Conjugated linoleic acid was identified as an active component with anti-aromatase activity [10]. Based on our preclinical data, a clinical trial of WBM is currently underway in postmenopausal, ER-positive breast cancer survivors to determine whether clinically detectable estrogen suppression can be achieved [14]. Although mukitake mushroom was reported to improve NAFLD, due to the observed anti-aromatase effect of WBM, there were clinical concerns that the resulting decrease in estrogen in women could exacerbate NAFLD in postmenopausal women. However, in a recent preclinical study we found that ovariectomized (OVX) mice fed a high fat diet with WBM powder (HFD+WBM) had relatively normal livers in terms of the size and appearance compared to mice fed a high fat diet (HFD) only. These observations, taken together with previous reports that show positive impacts of WBM intake on human health, led us to hypothesize that WBM may improve liver steatosis in postmenopausal women. To support our hypothesis, we also examined the effect of WBM on liver steatosis in the OVX mouse model and fatty acid metabolism in human hepatoma HepG2 cell line. The objective of the study was to gain insight into how dietary supplementation of WBM can prevent and/or ameliorate NAFLD in postmenopausal women.

## Materials and Methods

### Preparation of WBM diet

WBM were freeze-dried to steady moisture content, and then milled through a fine mesh. Microbiological and other analytical measurements were performed on pooled samples/batches to assure the quality of the WBM powder for research. The control HFD (45% (wt/wt) fat diet) and the HFD modified to contain freeze-dried WBM powder were produced and purchased from Research Diets, Inc (New Brunswick, NJ) (Table 1). For a proof-of-concept study to provide definitive data, the diet was composed of 120 g of WBM powder/kg of HFD, a similar dose as used previously to evaluate the effect of Mukitake mushroom diet for NAFLD [9]. Moreover, this therapeutic dose of WBM was shown to suppress estrogen-dependent and aromatase-positive breast cancer growth in our laboratory, using a nude mice model [10].

**Table 1:** Composition of experimental diets.

Ingredient	HF	HF-WBM
	(g/kg diet)	
Casein	165	165
L-Cystine	3	3
Corn Starch	224	171
Maltodextrin 10	132	132
Sucrose	100	100
Cellulose, BW200	50	50
Corn Oil	70	70
Lard	173	173
t-Butylhydroquinone	0.014	0.014
Mineral Mix S10022G	35	35
Vitamin Mix V10037	10	10
Choline Bitartrate	2.5	2.5
		
White button mushroom powder^a^	0	120

Powder was mixed to a modified AIN-93G diet enriched in fat (HF).

a Carbohydrate: 43.30%, fiber: 13.20% protein: 28.80%, fat: 4.50%

### Mouse experimental design

All animal research procedures were approved by the institutional animal care and use committee (IACUC) at City of Hope for assessment and accreditation of laboratory animal care, and were in accordance with NIH guidelines. Breeding stock strain C57Bl/6J was obtained from Jackson Labs. All animals were housed at the City of Hope Animal Resources Center in ventilated cage racks, had free access to water and were maintained on a 12 h light/dark cycle. All institutional guidelines for animal care and use were followed. The protocol for this study was approved by IACUC (protocol number: 08047).

To create a mouse model of postmenopausal women, seven-week old, female C57B1/6J mice were ovariectomized (OVX, n = 16). These OVX mice only produce estrogen from extragonadal sites and are used extensively as a model of postmenopausal women [4], [15]. Control mice (sham, n = 16) were subjected to sham operation survival surgery. The sham mice were subjected to the same general surgical procedure as OVX mice, except the removal of the ovaries. Mice were then divided into two dietary groups, with eight mice per group: HFD or HFD+WBM. The diets were started one week after surgery and continued for 3 months. The pair feeding design was used to control food intake. Food was weighed daily, and the control group given the same amount of food (by weight) one day later as the WBM group to ensure identical caloric intake between groups. At the conclusion of the experiment, mice were fasted for 4 hours before sacrifice. Blood of the mice was collected by cardiac puncture. After mice were euthanized, liver samples were collected.

### Pathological analysis

Livers were harvested from the mice and the wet weights measured. The tissues were fixed with 10% formalin overnight and then embedded in paraffin. Five-micrometer sections were cut and stained with hematoxylin and eosin, and examined by light microscope.

### Liver enzyme measurement

Blood samples were collected at the end of each experiment via cardiac puncture and centrifuged at 4000 rpm for 10 minutes to obtain serum. Serum alanine aminotransferase (ALT) levels were measured by Antech diagnostics company (Irvine, CA).

### Glucose tolerance test

Glucose tolerance tests were performed 2 months after starting the treatment diet. The mice were fasted with free access to drinking water for 18 hours prior to the test. Baseline glucose levels were recorded for each mouse. The mice were challenged with a 1.5 mg glucose/g body weight glucose load. The glucose levels pre, 30, 60, 120 and 180 min post-injection were measured using a glucometer (Bayer, Germany).

### Microarray analysis

For microarray analysis, total RNA was extracted from four liver specimens from OVX mice fed with HFD or from OVX mice fed with HFD+WBM using TRIzol reagent (Invitrogen, Carlsbad, CA). Synthesis and labeling of complementaryRNA (cRNA) targets, hybridization of GeneChips, and signal detection were carried out by the Microarray Core Facility at City of Hope. Briefly, The Affymetrix Mouse Gene 1.0 ST Array (Affymetrix, Santa Clara, CA) was used for microarray gene expression profiles. The microarray was carried out using Ambion's WT Expression kit (Life Technologies, Carlsbad, CA) and Affymetrix's GeneChip Terminal labeling system. Briefly, 100 ng of total RNA was used to start the first strand cDNA synthesis using an engineered random primer plus polyT7 promoter. After the 2^nd^ strand cDNA synthesis, the antisense cRNA (in vitro transcription) was carried using T7 RNA polymerase. Then, 10 µg of cRNA was used to start the 2^nd^ cycle of cDNA synthesis using random primers plus dUTP and dNTP mix. The single-strand cDNA was fragmented and then end-labeled with biotinylated nucleotides in the presence of terminal deoxynucleotidyl transferase (TdT) using Affymetrix WT Terminal Labeling kit. Five µg of labeled single-stranded cDNA was hybridized with Affymetrix Mouse Gene 1.0 ST array using the standard procedure as described previously [16] and the array was scanned using Affymetrix GeneChip Scanner 3000 7G.

### Statistical processing of Microarray data

Raw intensity measurements of all probe sets were corrected for background, normalized and converted into transcript-level expression measurements by using the IterPlier method in Affymetrix Power Tools (version 1.8.6). Quality assessment and statistical analysis of gene expression data were performed using the R/Bioconductor packages. To ensure the high quality of the microarray process, a set of quality assessment steps implemented in Bioconductor package Array Tools (http://www.bioconductor.org/packages/release/bioc/html/ArrayTools.html) were applied to the data. ArryTools was then used to identify the genes differentially expressed between OVX and OVX+WBM samples. Genes with significantly differentially expression were selected by use of cutoffs of p-value of 0.05 and 2-fold change in the level of expression. The genes showing altered expression were categorized and further investigated by enrichment analysis on the basis of their cellular components, biological processes, molecular functions, and canonical pathways using the Ingenuity Pathways Analysis (IPA)(Version 9.0)(Ingenuity) software. Data is MIAME compliant and the raw data has been deposited in NCBI Gene Expression Omnibus (GEO) with accession number of GSE31854.

### Ingenuity Pathway Analysis (IPA)

IPA is a Web-based software program that identifies the biological functions, pathways, and mechanisms most relevant to a given data set of genes. Information on individual genes is drawn from a large knowledge base of biological networks created from millions of publications, and the networks are drawn by the Functional Analysis feature of IPA based on the connectivity of the genes. To obtain a comprehensive view for the differentially expressed genes, core analysis was performed including network generation, functional analysis and canonical pathway analysis on the filtered (|Fold Change|>2, *P*<0.05) genes.

### Cell Culture

HepG2 cells were obtained from the American Type Culture Collection (ATCC) and grown in MEM containing 10% charcoal dextran treated fetal bovine serum (CD-FBS) in the presence of 100 U/ml penicillin and 0.1 g/l streptomycin. Cells were incubated at 37°C with 95% air and 5% carbon dioxide. All cells were kept below passage 20 and used in experiments during the linear phase of growth.

### Real-time PCR

The analysis was performed using RNA extracted from liver tissue (described under “Microarray analysis”) and from HepG2 cells. For cell culture studies, HepG2 cells were plated in 60 mm dishes at a density of 8×10^5^ cells/dish and treated with either DMSO or WBM extract. Twenty-four hours after treatment, the cells were collected for analysis of gene expression. Cells were washed with PBS, and Trizol reagent was used for total RNA isolation (Invitrogen). cDNA was synthesized with isolated RNA using reverse transcriptase III (Invitrogen). Real time PCR primers were as follows: mouse *β-actin*, 5'-CATTGCTGACAGGATGCAGAAGAAG-3' and 5'-CCTGCTTGCTGATCCACATCTGCT-3'; mouse fatty acid synthetase (*Fas*), 5'-TGGGTTCTAGCCAGCAGAGT-3' and 5'-AGACCGTTATGCCCAGACAG-3'; mouse fatty acid elongase (*Elovl6*), 5'-ACAATGGACCTGTCAGCAAA-3' and 5'-GTACCAGTGCAGGAAGATCAGT-3'; human *β-actin*, 5'-CACCAACTGGGACGACAT-3' and 5'-GCACAGCCTGGATAGCAA-3'; human *ELOVL6*, 5'-ACAATGGACCTGTCAGCAAA-3' and 5'-ATACCAGTGCAGGAAGATCAG-3'; human sterol regulatory element-binding protein 1 (*SREBP1c*), 5'-CCATGGATTGCACTTTCGAAGA-3' and 5'-GCTCAATGTGGCAGGAGGTG-3'; and human *FAS*, 5'-AGGCTGAGACGGAGGCCATA-3' and 5'-AAAGCTCAGCTCCTGGAGGT-3'. Reactions were run in triplicate on the iCycler iQ5 real time PCR detection system (Bio-Rad, Hercules, CA), and results were analyzed with the iQ5 software.

### Western blotting

HepG2 cells were seeded in 60 mm dishes at a density of 8×10^5^ cells/dish and treated with either vehicle control, Liver X receptor (LXR) agonist T0901317 (Cayman CHEMICAL, Ann Arbor, MI) (10 µM) or various concentrations of WBM extract for 48 hours. Proteins were isolated from HepG2 cells using passive lysis buffer (Promega, Fitchburg, WI). Liver tissues were collected at the end of experiment and then lysed using T-PER Tissue Protein Extraction Reagent (Thermo Scientific, Waltham, MA) according to the manufacturer's protocol. Briefly, liver tissues were weighed and homogenized in T-PER reagent containing protease and phosphatase inhibitor cocktail (Sigma-Aldrich, St. Louis, MO) using the T25 Basic tissue homogenizer (IKA Works, Inc., Wilmington, NC). For both tissue and cell lysis, samples were centrifuged at 10,000 X g for 5 minutes and the supernatant run on a 12% acrylamide gel, transferred to a nitrocellulose membrane and probed with antibodies to FAS (Abcam, Cambridge, MA), ELOVL6 (Abnova, Taipei City, Taiwan) or β-actin (Cell Signaling Technology, Inc., Danvers, MA). Bands were visualized via chemiluminescence using HRP-conjugated secondary antibodies and quantified using BioRad Quantity One software.

### Reporter gene assay

HepG2 cells were plated in 24-well plates at a density of 8×10^5^ cells/well and treated with either DMSO or WBM extract. To evaluate WBM effects on LXR activity, HepG2 cells were transiently transfected with the pCMX-VP16-hLXRα and LXR-responsive rCYP7A-DR-4x3-tk-LUC using the Lipofectamine Plus reagent system (Invitrogen) according to the manufacturer's protocol. 24 hours post-transfection, cells were treated with compound T0901317 (10 µM) alone or in combination with WBM extract (1, 2 and 5 µl/ml) for 24 hours. For evaluation of WBM effects on ER activity, HepG2 cells were transiently transfected with the pSG5-ER and pGL3 (ERE) 3-Luc using the Lipofectamine Plus reagent system (Invitrogen). 24 hours post-transfection, cells were treated with 17β-estradiol (E2) (Sigma-Aldrich) (0.1 nM) or with WBM extract (5 and 10 µl/ml) for 24 hours. Cell lysate was collected using passive lysis buffer and luciferase activity assayed on a TD 20/20 luminometer (Turner Designs, Sunnyvale, CA). Protein concentration was assayed using the BCA Assay (Thermo scientific). Data is expressed as relative luciferase unit/protein content.

### Production of Crude Mushroom Extract

As a control against the WBM investigation, we also did an analysis of other types of mushroom in a Mushroom Extract form. Mushroom extract was produced by chopping 60 g of fresh mushroom (WBM, shiitake mushroom (*Lentinus edodes*), enoki mushroom (*Flammulina velutipes*), or oyster mushroom (*Pleurotus ostreatus*)) and boiling the chopped mushroom in 500 ml water. The broth was filtered and centrifuged at 5,000 rpm for 30 minutes. Supernatant was rotor-evaporated until the final volume was 6 ml.

### Fractionation of WBM extract

Crude WBM extract was loaded to 5 g/60 ml capacity polyamide columns (Discovery DPA-6S SPE; Supelco). The flow through fraction was made from the extract that came out from the polyamide columns after crude WBM extract was loaded. Fractions were eluted by a step gradient (50 ml of each step) of increasing methanol to water. Flow through, the 0%, 20%, 40%, 60%, 80% and 100% methanol-water fractions were rotor-evaporated to dryness and then redissolved in 6 ml of 50% DMSO. Therefore, 60 g of WBM can produce 6 ml of each fraction.

### Placental microsomal aromatase assay

The aromatase assay was done by modification of a previous method [10]. The substrate, [1-β-^3^H] androstenedione, was dissolved in serum-free cell culture medium. Placental microsomes were prepared as 0.1 g/L in a potassium phosphate buffer (67 mM, pH 7.4) containing 20% glycerol, 0.5 mM dithiothreitol, and 0.25 M sucrose. The assay reaction mixture (225 µl), containing placental microsomes (2.5 µg), [1-β-^3^H] androstenedione (50 nM), progesterone (10 µM), and bovine serum albumin (0.1%) in potassium phosphate (67 mM, pH 7.4), with a sample solution in DMSO or H_2_O, was introduced in wells of a 96-well plate and preincubated at room temperature for 10 minutes; then, 25 µl of NADPH (3 mM) were added and the mixture was incubated at 37°C for 15 minutes. The reaction was terminated by addition of 50 µl of 20% trichloroacetic acid, and 250 µl of the reaction mixture was transferred to another well containing the charcoal-dextran pellet. The solution was thoroughly mixed and centrifuged (1,000 g, 5 minutes) to remove non-reacted substrate; an aliquot of the supernatant containing [^3^H] H_2_O as reaction product was counted in a Beckman Coulter LS 6500 Multi-Purpose Scintillation Counter. This modified assay method allows us to carry out 96 assays at the same time. Aromatase inhibition activity was calculated as the percentage of remaining activity from the reaction without WBM fractions. Analyses were carried out in triplicate and data were expressed as the mean ± SE.

### Statistical analysis

For comparison of the 2 diets (HFD and HFD+WBM) used in the *in vivo* experiment, a *t*-test was performed. The time-course experiment (body weight) was analyzed by 2-factor ANOVA. For multiple comparisons, analysis was followed by comparison of all treatment groups with the control group (Dunnett's test) or by comparison of all pairs of treatment (Tukey's test). Statistical significance was defined as *P*<0.05. All data are expressed as mean ± standard error (SEM). Data sets were analyzed for statistical significance using Prism GraphPad 4 software.

## Results

### WBM decreased liver weight and liver injury maker in OVX mice

**Figure 1:**
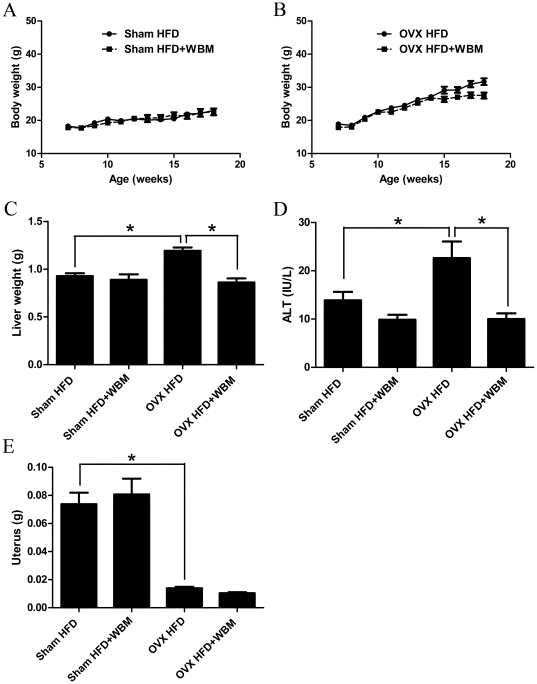
Effects of WBM feeding on body weight, liver weight, ALT serum levels and uterus weight. Seven-week old female C57B1/6J mice were ovariectomized. These mice are a model of postmenopausal women (OVX, n = 16). Control mice (sham, n = 16) were subjected to sham operation survival surgery. Both sham and OVX mice were fed a HFD (n = 8/per group) or HFD with WBM (n = 8/per group) diet for 3 months. Body weight was measured once a week. (A) Body weight measurement of sham mice. (B) Body weight measurement of OVX mice. (C) Liver weight. (D) ALT serum levels. (E) Uterus weight. Values are expressed as mean and standard error for 8 mice. * Statistical significance was defined as *P*<0.05.

No significant differences in body weight were observed between the control group fed the HFD and the group fed with HFD+WBM in both the sham and the OVX groups (sham: *P* = 0.8972, OVX: *P* = 0.0685) (Figure 1A and 1B). As expected, liver weight increased in HFD-fed OVX mice compared to HFD-Sham controls (*P*<0.01) and surprisingly, addition of WBM to the HFD inhibited this increase (*P*<0.01) (Figure 1C). In addition, serum ALT levels were higher in HFD-fed OVX mice than HFD-fed sham mice (*P*<0.05); this increase was suppressed by WBM (*P*<0.01) (Figure 1D). No significant differences in uterine weight were observed between HFD and HFD+WBM in both groups (Figure 1E).

### Fat accumulation in the liver was decreased in OVX mice after feeding mushroom diet

**Figure 2:**
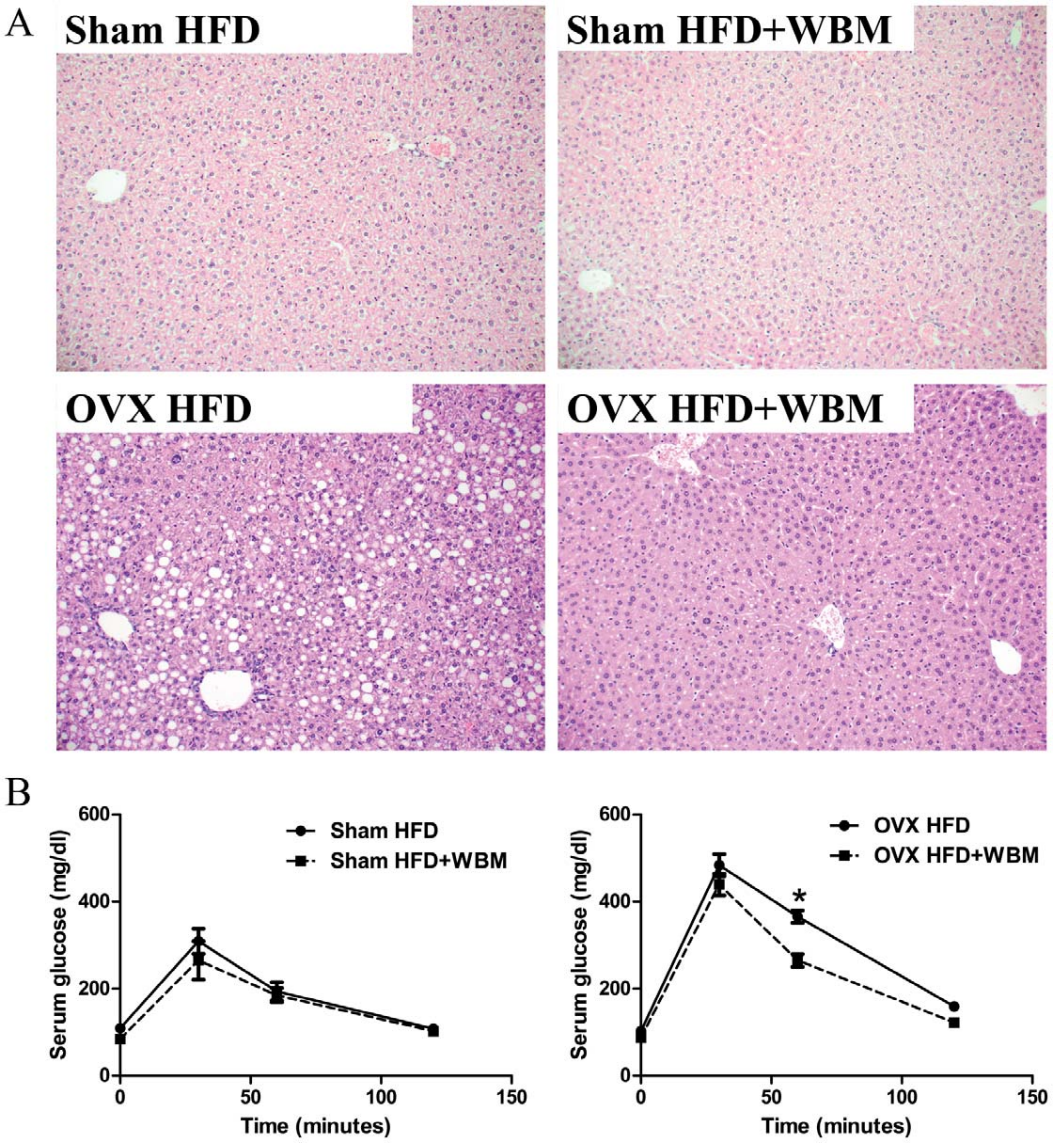
WBM intake decreased fat accumulation in the liver and improved glucose clearance ability in OVX mice. (A) Macroscopic appearance of liver from mice fed the HFD and HFD+WBM for 3 months in each sham and OVX group. The tissues were fixed with 10% formalin overnight and then embedded in paraffin. Five-micrometer sections were cut and stained with hematoxylin and eosin, and examined by light microscope. Representative H&E staining of livers from each group are shown. (B) Serum glucose concentration after glucose injection in mice fed with HFD or HFD+WBM diet for 2 months. The mice were challenged with 1.5 mg glucose/g body weight glucose load. The glucose levels pre, 30, 60, 120 and 180 min post injection were measured using a glucometer. Values are expressed as mean and standard error for 8 mice. * Statistical significance was defined as *P*<0.05.

Pathological examination of liver tissue showed fewer fat droplets (macrovesicular steatosis) in the cytoplasm of hepatocytes in mice fed HFD+WBM compared to those of mice fed only HFD. The fat droplets were concentrated in Zone III (near central veins) (Figure 2A).

### Increased insulin sensitivity in OVX mice fed with mushroom diet

After 2 months on the WBM diet, a glucose tolerance test was performed to evaluate circulating glucose concentration at each time point. In sham mice, base-line glucose concentrations were significantly lower in mice fed with HFD+WBM than mice with HFD (*P*<0.05). In OVX groups, the base-line serum glucose levels were not significantly different between mice with HFD and with HFD+WBM. Compared to the sham operation group, OVX mice showed higher serum glucose concentrations at 30 and 60 minutes post glucose injection (*P*<0.01). OVX mice on the WBM diet had improved glucose clearance at 60 minutes, compared to OVX mice fed with HFD only (*P*<0.01) (Figure 2B).

### Fatty acid biosynthesis pathways were down regulated in livers from mice fed with WBM

**Figure 3:**
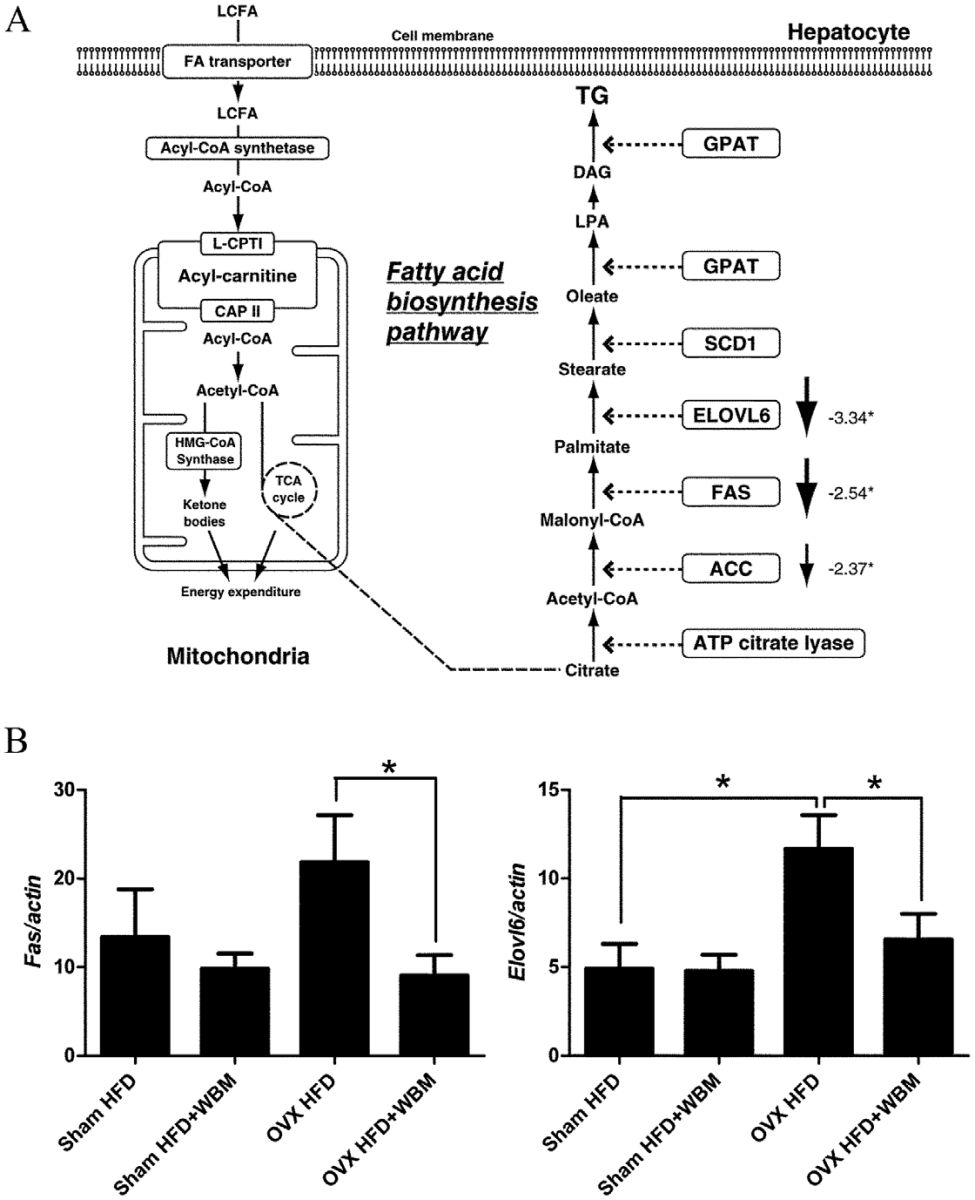
Changes of lipid metabolism in the liver identified by microarray analysis and confirmed using real time PCR. (A) Raw intensity measurements of all probe sets were corrected for the background, normalized and converted into transcript-level expression measurements by using the IterPlier method in Affymetrix Power Tools. The number indicated the fold changes of the gene expression in OVX+WBM compared to OVX. (B) *Fas* and *Elovl6* mRNA expressions in liver tissues from mice of each treatment groups (Sham HFD, Sham HFD+WMB, OVX HFD and OVX HFD+WBM) by real time PCR analysis. Gene expression was normalized with the *β-actin* housekeeping gene. Values are expressed as mean and standard error for 8 mice. * Statistical significance was defined as *P*<0.05 between HFD vs HFD+WMB treatment in each group (sham or OVX).

To study the protective mechanisms of WBM, genes that were regulated by the WBM diet in livers from OVX mice were analyzed for their functional grouping using IPA. The data set contained 20,234 unique genes. The top three networks of genes significantly altered by WBM identified by IPA in our data set are: (1) Small Molecule Biochemistry, Drug Metabolism, Lipid Metabolism; (2) Lipid Metabolism, Nucleic Acid Metabolism, Small Molecule Biochemistry; and (3) Organ Morphology, Reproductive System Development and Function, Small Molecule Biochemistry. Of these networks, two are involved in lipid metabolism and of prime interest to this investigation of the mouse liver phenotype: specifically, the levels of the genes involved in fatty acid biosynthesis pathway were significantly changed. The fatty acid biosynthesis pathway contains 7 genes, 4 of which passed our filter (Figure 3A). The Fisher's exact test was used to test whether the pathway of interest was over-represented in the filtered gene list. The fatty acid biosynthesis pathway gave a *P*-value of 1.05E-07, showing that this pathway was significantly changed by WBM intake. Specific genes in this pathway that were transcriptionally down regulated with statistical significance by WBM are: *Elovl6* (−3.34-fold, *P*<0.05), *Fas* (−2.54-fold, *P*<0.05), *Acc* (−2.37-fold, *P*<0.05) and *ATP citrate lyase* (−2.33 fold, *P*<0.05). The microarray analysis results were validated by real time PCR using RNA extracted from all treatment groups (sham, sham+WBM, OVX and OVX+WBM). Increased *Elovl6* expression was shown in OVX mice fed with HFD compared to sham mice with HFD (*P*<0.05). *Fas* expression was increased in OVX mice fed with HFD compared to sham mice with HFD; however it did not reach statistical significance. Results showed that addition of WBM to the HFD significantly decreased *Fas* and *Elovl6* expressions in the livers of OVX mice (*P*<0.05), which is in agreement with the results of our microarray analysis (Figure 3B).

### WBM extract downregulated FAS and ELOVL6 expressions in HepG2 cells

**Figure 4:**
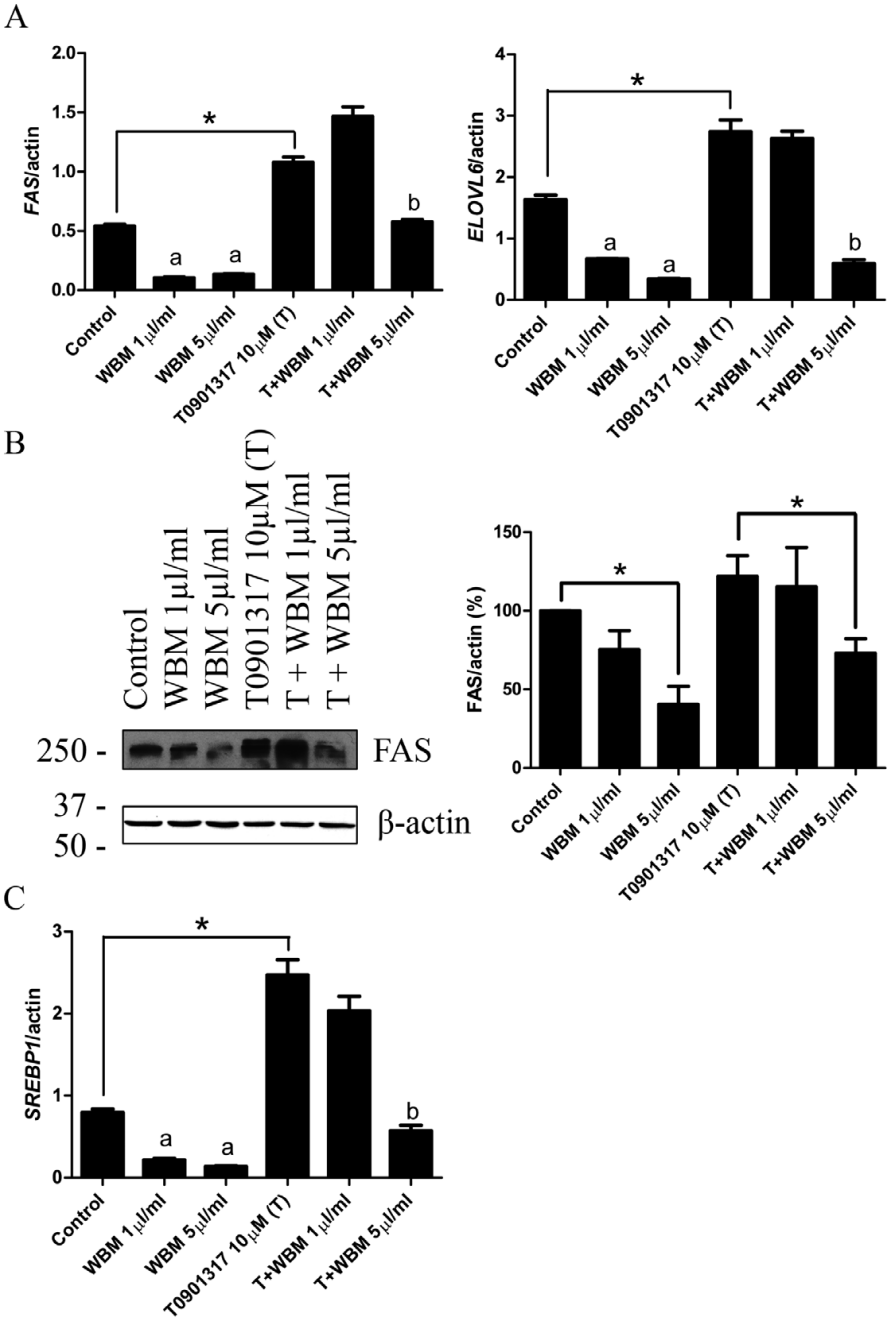
Changes in the mRNA and protein levels of genes related to fatty acid synthesis pathway in HepG2 cells treated with WBM extract. Cells were incubated with vehicle control (Ethanol), T0901317 (10 µM) and/or WBM extract (1 and 5 µl/ml) for 24 hours for RNA and 48 hours for protein, respectively. (A) *FAS* and *ELOVL6* mRNA levels. Gene expression was normalized with the β-actin housekeeping gene. (B) FAS protein expressions were determined via western blotting. Bar graphs indicate quantification of three separate blots using Quantity One software. Values are expressed as mean and standard error. (C) *SREBP1* mRNA levels. We analyzed the data for each dose separately by ANOVA, followed by Tukey's multiple comparison test. Statistical significance was defined as * *P*<0.05 compared indicated treatment, a; *P*<0.05 compared to control, b; *P*<0.05 compared to T0901317 treatment.

To further define the effect of crude WBM extract, the expression of *FAS* and *ELOVL6* was measured in a human hepatoma cell line, HepG2. *FAS* and *ELOVL6* gene expressions were significantly decreased in cells treated with WBM extract in a dose dependent manner after 24 hours of treatment, in two doses (1 and 5 µl/ml) (*P*<0.01). To confirm the results from HepG2 cells, we performed real time PCR to evaluate the levels of *ELOVL6* mRNA using another cell line, Huh7 cells. Huh7 is a well differentiated hepatocyte derived cellular carcinoma cell line. Since a similar degree of inhibition of *ELOVL6* mRNA expression was found in Huh7 cells by WBM extract compared to HepG2 cells (data not shown), we have performed additional experiments using HepG2 cells. Treatment of the compound T0901317, which is an agonist for LXR, increased the mRNA levels of these genes significantly, compared to controls (*P*<0.01). T0901317-induced *FAS* and *ELOVL6* mRNA levels were decreased in the HepG2 cells treated with WBM extract (5 µl/ml) (*P*<0.01) (Figure 4A). FAS protein expression was also significantly decreased in the cells treated with WBM extract (Figure 4B). Notably, the changes in *FAS* and *ELOVL6* mRNA expression paralleled the changes of *SREBP1c* expression in HepG2 cell lines with each treatment (Figure 4C).

### Identification of the active fractions

**Figure 5:**
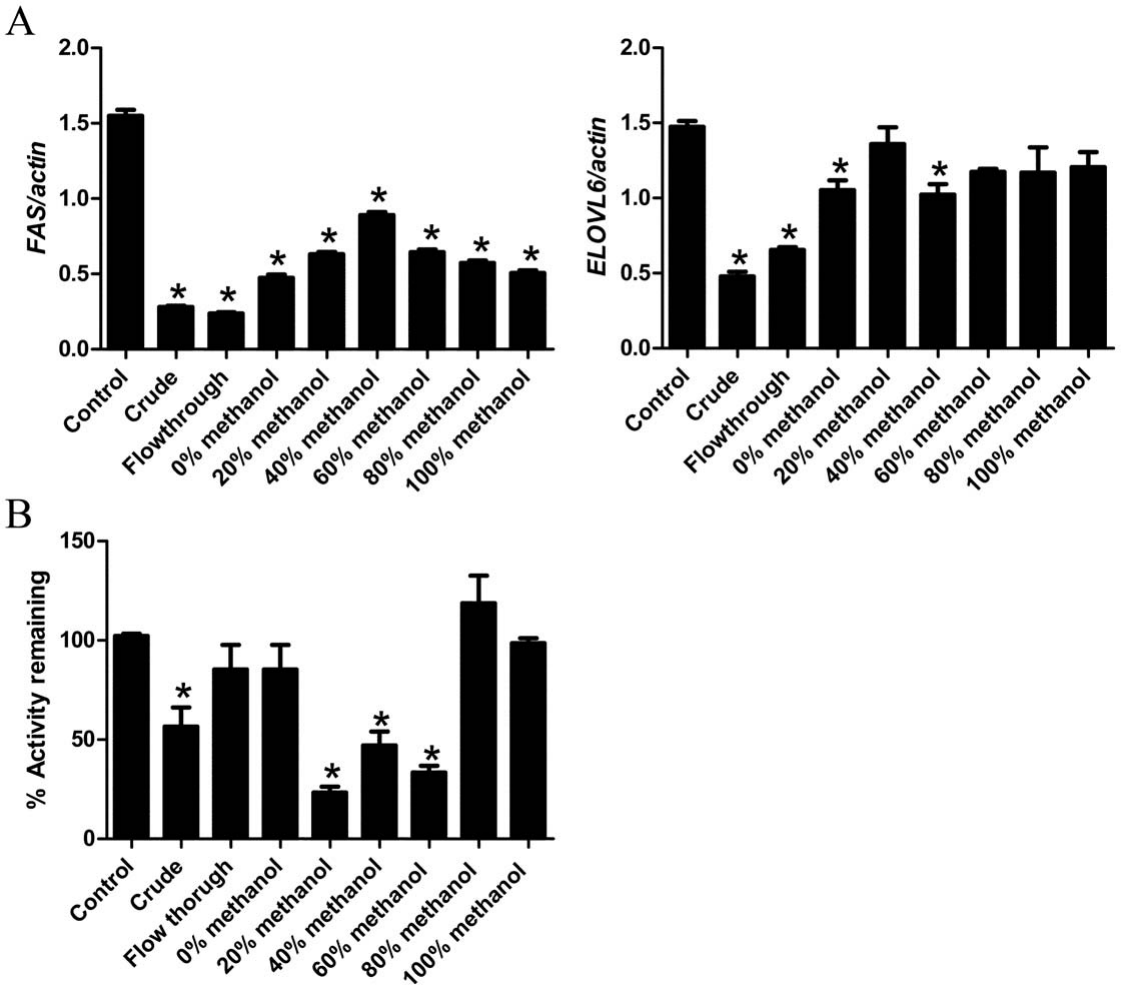
Effects of methanol fractions from WBM extract on *FAS* and *ELOVL6* gene expressions and aromatase activity in HepG2 cells. Cells were incubated with each methanol fraction from WBM extract for 24 hours. (A) *FAS* and *ELOVL6* gene expression was normalized with the β-actin housekeeping gene. (B) Microsome assays were performed to evaluate anti-aromatase effects of WBM using the substrate, [1-β-^3^H] androstenedione. Activity was calculated to measure supernatant containing [^3^H] H_2_O as reaction product, and then was counted in a Scintillation Counter. Aromatase inhibition activity was calculated as the percentage of remaining activity from the reaction without mushroom fractions. Analyses were carried out in triplicate and data were expressed as the mean ± SE. We analyzed the data for each treatment by ANOVA, followed by comparison of all treatment groups with the control group (Dunnett's test). Statistical significance was defined as *P*<0.05.

After separation of WBM extract based on methanol concentration, the expression of *FAS* and *ELOVL6* genes was assayed by real time PCR. We analyzed the data for each treatment by One-way ANOVA, followed by comparison of all treatment groups with the control group (Dunnett's test). The *FAS* expression was significantly inhibited (*P*<0.01) by all fractions, with the flow through fraction providing the strongest inhibition, similar to that of the crude fraction. Crude and flow through fractions significantly inhibited *ELOVL6* expression in HepG2 cells compared to the control treatment (*P*<0.01). The 0% methanol ( =  water fraction) and 40% methanol fractions had moderate inhibitory effects (*P*<0.05) (Figure 5A). The inhibitory effect of WBM extracts on aromatase activity was also evaluated. The flow through fraction did not show any inhibition; however, the 20–60% fractions provided the strongest inhibitory effect among all fractions (Figure 5B). These latter results are consistent with previous reports, which showed that the active “anti-aromatase” compound in this 20–60% fraction was conjugated linoleic acid (CLA) [10]. While we have not yet identified the chemicals that inhibit the fatty acid biosynthesis pathway, the current results suggest that CLA is not the active component in the inhibition of the fatty acid biosynthesis pathway in liver.

### WBM extract modulates Liver X receptor (LXR) function

**Figure 6:**
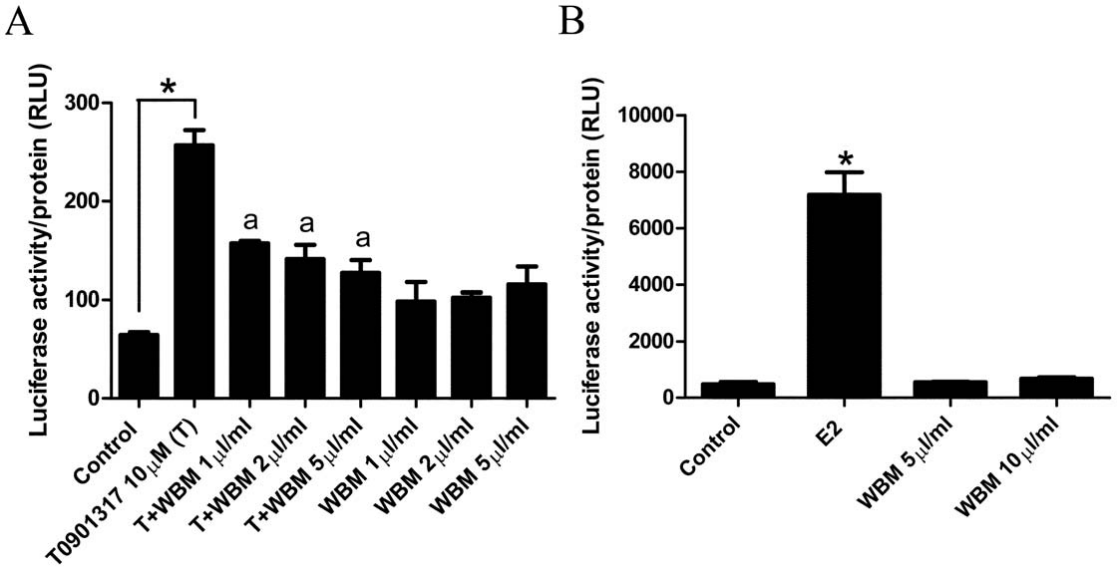
LXR and ER activity in HepG2 cells treated with WBM extract. (A) HepG2 cells were transfected with the pCMX-VP16-hLXRa and LXR-responsive rCYP7A-DR-4x3-tk-LUC, and following were incubated with vehicle control (ethanol), T0901317 (10 µM) and/or WBM extract (1, 2 and 5 µl/ml) and assayed for luciferase activity. (B) For evaluation of WBM effects on ER activity, HepG2 cells were transiently transfected with the pSG5-ER and pGL_3_(ERE)_3_-Luc. 24 hours posttransfection, cells were treated with E2 (0.1 nM) or with WBM extract (5 and 10 µl/ml) for 24 hours. Data is expressed as relative luciferase unit/protein content. Values are expressed as mean and standard error. For LXR activity, we analyzed the data for each dose separately by ANOVA, followed by Tukey's multiple comparison test. * *P*<0.05 compared indicated treatment, a; *P*<0.05 compared to T0901314 treatment. For ER activity, we analyzed the data for each treatment by ANOVA, followed by comparison of all treatment groups with the control group (Dunnett's test). Statistical significance was defined as *P*<0.05.

The IPA analysis showed that the canonical pathway for LXR/RXR activation was significantly inhibited in the livers of OVX mice fed with WBM (*P* = 1.36E–02) compared to OVX mice fed with HFD. To evaluate the direct effects of WBM extract on LXR activation, an LXR-luciferase reporter assay was performed. The LXR agonist T0901317 significantly increased the luciferase activity in HepG2 cells transfected with the pCMX-VP16-hLXRα and the LXR-responsive rCYP7A-DR-4x3-tk-LUC plasmid (P<0.01). The LXR-luciferease activity was significantly decreased when cells were treated T0901317 together with WBM extract in a dose dependent manner (*P*<0.01). The WBM extract treatment itself increased LXR-luciferase activity modestly, however this increase was not significant (*P*>0.05) (Figure 6A). These results suggest the presence of LXR antagonist(s) in the WBM extract. Using an ER-positive MCF7 cell line, the ER-luciferase reporter activity was significantly increased by treatment of E2 (*P*<0.01); however, treatment with WBM extract did not affect ER-luciferase activity (Figure 6B), indicating that the extract does not contain estrogen-like chemicals and ER is not involved in the WBM-mediated protection of the development of NAFLD.

### Comparison of anti-NAFLD effects of various species of mushroom

**Figure 7:**
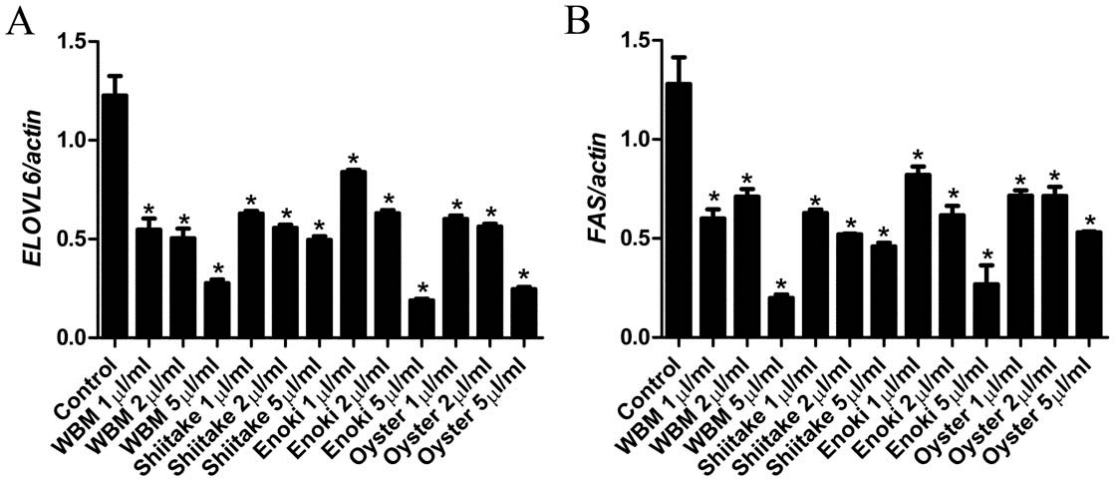
Comparison of the effects of various species of mushroom on the fatty acid synthesis pathway. (A) *FAS* and (B) *ELOVL6* gene expressions in HepG2 cells treated with various species of mushroom extract (WBM, shiitake mushroom, enoki mushroom and oyster mushroom) for 24 hours. Real time PCR analysis was performed for the gene, *ELOVL6 and FAS*. Gene expression was normalized with the *β-actin* housekeeping gene. Values are expressed as mean and standard error. We analyzed the data for each dose separately by ANOVA, followed by Tukey's multiple comparison test. Statistical significance was defined as *P*<0.05 compared to control.

The extracts from enokitake mushroom, shiitake mushroom, oyster mushroom, and WBM were tested for their ability to suppress the fatty acid biosynthesis in HepG2 cells. Dose-dependent inhibition of *FAS* and *ELOVL6* gene expression was found in all four mushroom species compared to untreated controls (*P*<0.01). WBM showed greater inhibition of *FAS* expression compared to oyster mushroom (*P*<0.05) but not to other mushrooms at dose of 5 µl/ml; there were no significant differences in activity among four mushroom species at doses of 1 and 2 µl/ml (Figure 7A). *ELOVL6* expression was inhibited better by the extracts of enokitake mushroom, oyster mushroom and WBM compared to shiitake mushroom at the highest dose of 5 µl/ml (*P*<0.05). At the low dose (1 µl/ml), WBM had the highest inhibition (*P*<0.05) (Figure 7B).

## Discussion

The results of this study suggest that WBM has a protective effect against liver steatosis in the OVX mice, as a model for postmenopausal women. The protective effect observed in our mouse model with regard to liver steatosis was found to be mediated though the suppression of fatty acid biosynthesis, as indicated by our microarray data. In addition, WBM diet improved insulin resistance observed in OVX mice fed with HFD. Our finding is important since several papers have reported that mushroom may have anti-atherogenic effects through modulation of lipid profiles [12], [17], [18]. Although WBM is the most common edible mushroom in the USA, to our knowledge, this is the first study to show WBM has a protective effect against liver steatosis in the mouse model of postmenopausal women.

NAFLD is a disease more frequent in postmenopausal women than premenopausal women [19]. Aromatase knockout mice, which are deficient in estrogen pathway, develop hepatic steatosis. Moreover, hepatic steatosis was diminished in animals treated with E2 [20]. Patients with a mutation of the aromatase gene showed liver steatohepatitis, and estrogen treatment resulted in the improvement of liver steatohepatitis [21]. These reports confirm that estrogen has an important protective role against the development of NAFLD. Tamoxifen is an antiestrogen used widely in the treatment and chemoprevention of breast cancer. Hepatic steatosis and non-alchoholic steatohepatitis have been reported in patients treated with tamoxifen [22]. Therefore, fat accumulation in liver is an important clinical concern in postmenopausal women, especially those have anti-estrogenic therapy. OVX mouse is the established model for postmenopausal women. Studies in rodents showed that OVX promotes obesity and its metabolic complication [4], [15]. As a model of postmenopausal women, our studies using OVX mice confirm a high fat diet promotes liver steatosis, and a WBM-containing diet can alleviate liver steatosis. The serum transaminase (ALT) level was measured, as a sensitive method used in the detection of liver statosis in clinical diagnosis [1]; it is also linked to liver fat content [23]. Our results showed that serum ALT levels were significantly lowered in OVX mice fed with WBM, to a similar level as in sham mice. It is worthwhile to note that the WBM did not change mouse body weight, but did have an impact on liver steatosis. This suggests that WBM affects liver function specifically. Several rodent models have shown that decreasing hepatic triglyceride pools correlates with improved insulin sensitivity [1]. Also, several lines of evidence suggest that lipid metabolites in the lipogenesis pathway in liver are determinants for the development of insulin resistance [1]. Thus, we performed glucose resistance tests to evaluate glucose clearance ability in OVX mice fed with WBM diet. Our data showed that a WBM diet could improve steatosis-associated insulin resistance in the OVX mice.

Our group previously reported that WBM extract inhibited breast cancer cell growth *in vitro* and *in vivo* through inhibition of the aromatase enzyme [10]. Our studies have revealed that the chemicals that suppress fatty acid biosynthesis in liver are different from those that inhibit aromatase activity. Furthermore, the WBM extract does not have any estrogen-like activity, indicating that the protective mechanism of WBM against liver steatosis is not estrogen-mediated. Uterus weights were not changed in both sham treated and OVX mice fed with WBM diet compared to those fed with HFD only diet (Figure 1E). These results suggest that WBM did not significantly change circulating estrogen levels in both sham treated and OVX mice. Based on our current data, WBM may have a beneficial effect on the liver in postmenopausal women even at low estrogen conditions, particularly those women who are under anti-estrogen therapy.

Microarray analysis revealed that multiple enzymes involved in the fatty acid biosynthesis pathway were significantly down regulated in the liver of OVX mice fed with a diet containing WBM. *Fas* and *Elovl6* were most down regulated by WBM treatment. FAS plays an important role in the synthesis of fatty acids via *de novo* lipogenesis. FAS catalyzes the last step in the fatty acid biosynthetic pathway [1]. ELOVL6 is a rate-limiting enzyme for the elongation of saturated and monounsaturated long chain fatty acids. ELOVL6 is expressed in the liver and its mRNA levels are up-regulated in obese animals [24]. Thus, researchers have evaluated the therapeutic potential of ELOVL6 inhibitors for metabolic disorders [25]. HepG2 is a human liver-derived hepatoma cell line, and many liver-specific functions are maintained in this cell line [26]. Since the expression of FAS and ELOVL6 was decreased after WBM extract treatment *in vitro* in the HepG2 cells, the active components in WBM are probably not metabolites of biological conversion in an intact animal. Moreover, multiple enzymes in the fatty acid biosynthesis pathway are down-regulated by WBM extract, suggesting common mechanisms may exist to regulate multiple enzymes. LXR has been reported to control genes that encode proteins involved in *de novo* lipogenesis, including *FAS* and *ELOVL6*. Moreover SREBP1c is regulated through LXR [1]. Oral administration of the synthetic LXR agonist, T0901317, to C57BL/6 mice resulted in increased hepatic triglyceride levels, leading to liver steatosis [27]. Our results showed that WBM extract down-regulated T0901317-induced LXR receptor activity in the HepG2 cells, suggesting that WBM extract mainly modulates LXR function in liver.

We also evaluated the effects of several types of mushrooms *in vitro* using the HepG2 cells. All of the mushroom extracts showed a dose-dependent inhibition of the expression of *FAS* and *ELOVL6*. Although significant efforts have been made, we cannot yet identify the phytochemicals that are responsible for the “anti-liver steatosis” effect of mushrooms. Our previous report showed that CLA is an active component with anti-aromatase activity (to reduce estrogen) [10]. The CLA-containing fractions did not show strong inhibition of *ELOVL6* and *FAS* gene expression in HepG2 cells. Moreover, treatment with CLA did not decrease liver weight and accumulation of fat in the livers of OVX mice [28]. These results suggest that CLA is not the active component in the inhibition of the fatty acid biosynthesis pathway in liver. Phytosterols, such as oxysterols, have been found to be ligands of LXR [29]. Ergosterol peroxide is an oxysterol that can be produced by a hydrogen peroxide-dependent enzymatic oxidation of ergosterol [30]. Ergosterol is an important sterol in WBM and is shown to be a vitamin D2 precursor [31]. However, ergosterol was not able to inhibit the expression of *ELOVL6* and *FAS* in HepG2 cells in our *in vitro* studies (data not shown), suggesting that the active chemical(s) may be another sterol or oxidized sterol.

In order to determine whether our results have a potential application to women's health, the human equivalent of doses used in this study needs to be determined. The average WBM intake in mouse studies is approximately 260 mg/day (average food intake 2.2g/day, WBM diet 120 g powder/kg diet). The human equivalent dose (based on body surface area) [32] is equal to 42 g WBM powder for a 60 kg person. It is important to point out that this would be a therapeutic dose to achieve the development of NAFLD. This is a proof-of-concept study; therefore, the liver protective effect could be achieved probably with lower levels of WBM for women consuming a regular diet. Moreover, drug discovery from natural products has been one of the main approaches to develop new drugs. We are continuing our efforts in the functional characterization of active components in WBM.

In summary, NAFLD is a widespread problem associated with many metabolic disorders that has become increasingly prevalent in our society. Postmenopausal women have the highest risk of developing NAFLD, compared to premenopausal women and men. The incidence would be even higher for those postmenopausal women under anti-estrogen therapy and with a high BMI. The pathogenesis of NAFLD is complicated and involves multiple pathways. Single protein target drugs are not successful in the treatment of NAFLD, and there is currently no generally accepted therapeutic intervention for NAFLD. To date, the only effective treatment of NAFLD is a general lifestyle change, including diet, weight reduction and exercise. Our results showed that WBM intake may be a viable dietary choice to prevent liver steatosis, which is an early reversible stage of NAFLD in postmenopausal women. WBM is widely available and inexpensive. If this effect could be demonstrated in humans, postmenopausal women would have a natural option for avoiding liver steatosis and its associated conditions. This prevention of early stage of the disease leads to reduce the risk of NAFLD. Furthermore, at the same time, WBM would suppress aromatase to decrease risk of breast cancer.
